# Patient Perspectives Towards Artificial Intelligence in Heart Failure Care

**DOI:** 10.7759/cureus.98378

**Published:** 2025-12-03

**Authors:** Sirindhra Suepiantham, Ravish Katira

**Affiliations:** 1 Cardiology, Mersey and West Lancashire Teaching Hospitals NHS Trust, Liverpool, GBR

**Keywords:** artificial intelligence, cardiology, heart failure, questionnaire, telemedicine

## Abstract

Background

Artificial intelligence (AI) tools are increasingly being developed to support the management of chronic diseases, including heart failure (HF). Little is known about the views of patients with HF, a population typically older, with high comorbidity, and familiar with close clinician relationships. This study aims to investigate HF patients’ perception of AI integration into HF care to help guide a patient-centred adoption.

Methods

We conducted a cross-sectional survey on consecutive patients with HF with reduced ejection fraction attending follow-up at a clinic. The questionnaire assessed satisfaction with current care, trust in cardiologists, and attitudes toward AI involvement in diagnosis and treatment decisions. Responses were collected on Likert scales and analysed using ordinal and binary logistic regression to test associations with age, sex, education level, smartphone ownership, symptom burden and prior experience with AI.

Results

A total of 110 patients completed the questionnaire. Attitudes towards AI were mixed. While 38.1% were happy for their doctor to use AI's help in treatment decisions, support fell significantly to 18.2% when AI acted without physician input and to 21.8% when AI remotely adjusted treatment with fewer in-person visits. Even when described as outperforming doctors, half of the patients remained uncomfortable. In direct comparisons, 80.9% preferred cardiologist diagnoses, 84.6% preferred cardiologist treatment plans, and 97.3% would trust their cardiologist over AI in cases of disagreement. No association was found with age, sex, or education. Smartphone ownership, however, predicted greater acceptance of remote AI adjustments.

Conclusion

HF patients report high satisfaction with care and strong trust in cardiologists, with more cautious attitudes toward AI than seen in general patient populations. Smartphone ownership, rather than demographics or specific experience with AI, predicted openness to AI. Preserving clinician oversight and designing accessible AI tools will be key to equitable adoption in HF care.

## Introduction

Heart failure (HF) remains a leading cause of morbidity, mortality, hospitalisation, and healthcare expenditure worldwide, with a prevalence of 1-3% in the adult population and rising steadily as populations age and survival from acute cardiac events improves [1]. HF patients commonly have cardiac and non-cardiac comorbidities such as atrial fibrillation, diabetes, and chronic kidney disease, which can increase mortality and HF hospitalisation [1]. Furthermore, social determinants such as income and education level can contribute to the risk of HF-related death [2]. With so many dynamic factors influencing HF, optimal HF management therefore requires regular follow-up and shared decision-making between patients and clinicians to monitor symptoms, adjust pharmacotherapy, and implement lifestyle measures [3].

Recently, artificial intelligence (AI)-enabled tools have emerged to assist clinicians in diagnosis, risk prediction, and treatment optimisation. Machine-learning models can facilitate early detection of HF and phenotype complex syndromes and predict outcomes by analysing multidimensional data drawn from imaging, electronic health records, laboratory testing, and wearable devices [4-6]. AI-driven applications are also increasingly available directly to patients, aiming to support self-monitoring and avert hospitalisation through early detection of congestion or decompensation using smartphones or connected sensors [7]. Examples include commercially available platforms such as HearO and Linea, which have shown promising early results and the potential for substantial cost savings [7-9].

Despite these technical advances, the clinical impact of AI ultimately depends on patient’s acceptance and willingness to use them. Surveys of patients and the public suggest broad openness to AI in healthcare for administrative tasks, symptom assessment, and remote monitoring, but persistent reluctance when AI is applied to diagnosis or autonomous treatment decisions [10]. Common concerns include loss of human contact, accountability for errors, and data privacy [11]. Acceptance is typically higher when AI is framed as augmenting rather than replacing clinicians [11].

Prior studies have also indicated that age, sex, education level, health literacy, and prior digital experience can influence willingness to engage with AI-enabled care [12-14]. For example, older adults often express more caution, whereas individuals with greater digital literacy may exhibit higher trust and adoption rates [12-14]. Furthermore, cultural differences and healthcare systems may impact intention to use health technologies [15,16]. Conceptually, the Technology Acceptance Model (TAM) suggests that perceived usefulness and perceived ease-of-use underpin acceptance of digital tools and, together with additional psychological and contextual factors, can predict why people accept or reject a technology [17,18]. Similarly, the Unified Theory of Acceptance and Use of Technology (UTAUT), which synthesises the TAM and other technology acceptance models, have the key determinants of performance expectancy, effort expectancy, social influence, and facilitating conditions as predictors for whether individuals will adopt a technology [19]. Age, gender and experience with technology are moderators that further influence the key determinants and thus how groups will respond to technology [19]. The TAM and UTAUT model are widely used in health technology and AI acceptance research. For instance, a study by Zin et al. concluded that perceived usefulness and ease of use, as well as facilitating conditions, have a significant impact on participants’ attitudes towards using a smart health watch [20]. Zhu et al. concluded that TAM and UTAUT constructions including social influence and perceived trust had positive effects on use intention of mobile health application [21]. Schretzlmaier et al. suggest that UTAUT constructs are suitable for predicting acceptance of digital health tools; however, the additional constructs of trust and perceived disease threat should be added [22].

To our knowledge, no published study has specifically examined the attitudes of HF patients toward AI-enabled care, despite their unique clinical needs. This population is typically older, frequently interacts with the healthcare system, and carries multiple comorbidities, factors that may influence AI acceptance in distinctive ways [23]. Trust is particularly crucial in chronic diseases like HF as longitudinal management and high-risk therapy adjustments are required. Interpersonal dynamics, empathy, and the accountability associated with clinicians remain central to patient trust, elements that are not easily replicated by AI [24]. Understanding the perspectives of people living with HF is, therefore, essential as AI tools and remote monitoring platforms expand in this domain to ensure their effective development, equitable deployment, and meaningful adoption.

This study addresses the lack of understanding of HF patients’ perception of AI, specifically in the context of a UK public healthcare system (National Health Service). It is focused specifically on patients with heart failure with reduced ejection fraction (HFrEF) rather than other HF phenotypes (such as HF with preserved ejection fraction (HFpEF)) as HFrEF has a well-established evidence base for prognostic therapies, a higher risk of recurrent decompensation, and a greater need for frequent medication titration compared with HFpEF [25]. Guidelines for medical therapy for HFrEF often call for sequential, multi-drug optimisation with repeated monitoring of symptoms, renal function, blood pressure, and tolerability [3]. Consequently, patients with HFrEF typically interact more frequently with healthcare services and may be particularly affected, positively or negatively, by emerging AI-enabled tools designed to support disease monitoring and treatment adjustment. The study also aims to examine whether demographic characteristics, symptom burden, digital literacy (smartphone use) and previous experience with AI impact attitudes.

## Materials and methods

Study design

In this cross-sectional study, all consecutive patients with a diagnosis of HFrEF attending a follow-up HF clinic in St Helens, England, between August 1, 2024 and March 31, 2025 were approached and invited to participate. Patients received a written explanation of the purpose of the study for quality improvement and verbal consent was obtained prior to questionnaire distribution. A written explanation of AI was provided to all patients to read with the questionnaire. No formal sample size calculation was performed because this study was designed as an exploratory, single-centre quality improvement survey. The sample reflects all consecutive eligible patients attending the clinic during the study period.

Questionnaire

To create the questionnaire, the authors conducted a literature review using PubMed (Appendix). The questionnaire contained five questions which gathered demographic data (sex, age, education level, smartphone ownership, and experience with AI), nine questions (Q1-Q9) which used the Likert scale (1-5) to assess patients perception of their current care and happiness for level of AI involvement, and one question (Q10) which made patients choose whether they would trust a cardiologist or AI if the two disagreed on treatment plan. The Participant Information Sheet and Questionnaire can be found in the Appendix.

Inclusion criteria

Patients who had a diagnosis of HFrEF attending an outpatient HF clinic in St Helens between August 1, 2024 and March 31, 2025 were included in the study.

Exclusion criteria

Patients who lacked capacity, patients who could not read English, and new HF patients (those who were attending the HF clinic for the first time) were excluded from the study. Responses from patients who did not complete the full survey were excluded from analysis.

Statistical analysis

Patients’ answers on paper questionnaires were promptly transferred into a password-protected Microsoft Excel spreadsheet on a secure hospital computer and imported into IBM SPSS Statistics for Windows, Version 29 (Released 2023; IBM Corp., Armonk, New York, United States) for statistical analysis. All variables were summarised using descriptive statistics (counts and percentages). Likert-scale responses were analysed using ordinal regression. Age, education level, and experience with AI were included as continuous covariates, while sex (male/female) and smartphone ownership (yes/no) were included as categorical covariates. Patients’ symptom burden (Q1), satisfaction with current follow-up (Q2), and confidence in their cardiologist (Q3) were also examined for associations with other Likert-scale questions using ordinal regression with the same covariate adjustments. Binary logistic regression was used for Q10, with the same adjustment set.

To compare paired responses to questions regarding different levels of AI involvement (Q4 vs. Q5 vs. Q6), McNemar’s test was applied after collapsing responses into two categories: “unhappy” and “neutral/happy”. A significance level of p<0.05 was considered statistically significant.

No correction for multiple comparisons was applied, as the analyses were exploratory and intended to identify potential associations rather than to test predefined hypotheses. Multicollinearity among predictors was evaluated using variance inflation factors (VIFs) generated via linear regression models containing the same independent variables used in the ordinal and logistic regression analyses. All VIF values were <1.5 (range 1.108-1.456), indicating no evidence of problematic multicollinearity.

Compliance with ethics guidelines

This project was approved by Whiston Hospital’s QI-Clinical Audit Department, approval number S332 23 24. Verbal consent was obtained from patients prior to questionnaire distribution. As an anonymous low-risk survey, the QI-Clinical Audit Department deemed that written consent was not required. Tenets of the Declaration of Helsinki of 1964 were adhered to [26]. Paper questionnaires were immediately discarded in line with the trust’s guidelines for confidential waste management once responses were transferred to a secure hospital computer. Data was stored in line with the hospital’s and QI-Clinical Audit Department’s guidelines.

## Results

There was a total of 136 patients eligible for the survey, with 110 patients completing the full questionnaire. Their demographics are summarised in Table 1. No one declined to participate. Responses from incomplete questionnaires (n=26) were omitted from further analysis. Frequency tables for Q1-Q10 are available in the Appendix.

**Table 1:** Patient demographics and perceived symptom burden. Patients reported demographic information. Ages were collated into groups. Patients self-reported the level of education and experience with AI based on preset options. As a measure of symptom burden, patients report how troublesome they find their heart failure using Likert scale 1-5. Percentages add up to >100 due to rounding.

Sex	
Male	72 (65%)
Female	40 (36%)
Age	
<60	9 (8%)
60-69	25 (23%)
70-79	49 (45%)
80-89	25 (23%)
90-99	2 (2%)
Level of Education	
Primary/secondary	75 (67%)
Diploma	20 (18%)
Bachelor’s degree	8 (7%)
Postgraduate degree	8 (7%)
Smartphone ownership	
Owns a smartphone	79 (71%)
Does not own a smartphone	33 (29%)
Experience with AI	
Work or study in a field related to AI	2 (2%)
Understand how AI functions	19 (17%)
Have researched the term AI	3 (3%)
Have heard of the term AI	52 (46%)
Do not know AI	36 (32%)
Q1: How troublesome is your heart failure?	
1 - Not troublesome at all	15 (14%)
2	19 (17%)
3	26 (24%)
4	25 (23%)
5 - Very troublesome	25 (23%)

Patients’ satisfaction with current service (Q2, Q3)

Overall, 93.6% of patients reported being satisfied (Q2 = 4 or 5) with the follow-up they are currently receiving for their HF, while 4.5% were neutral (Q2 = 3). The same number of patients (93.6%) also expressed confidence in their cardiologist’s treatment plan (Q3), while 4.5% were neutral. There was no statistically significant association between age, sex, or education level and patient satisfaction or confidence.

Ordinal logistic regression showed a graded association between symptom burden and satisfaction. Compared with patients whose HF symptoms were “not troublesome at all” (Q1 = 5), the odds of reporting greater satisfaction were substantially lower among those with very troublesome symptoms (Q1 = 1: OR = 24.9, 95% CI 1.38-449.8, p = .029), troublesome symptoms (Q1 = 2: OR = 11.2, 95% CI 1.27-98.9, p = .030), and slightly troublesome symptoms (Q1 = 4: OR = 17.3, 95% CI 1.60-188.9, p = .019). The moderate category (Q1 = 3) trended in the same direction but was not statistically significant (OR = 7.74, 95% CI 0.82-72.9, p = .074). The wide confidence interval reflects the small number of patients in some symptom-burden categories. This suggests that the magnitude of the association should be interpreted cautiously. There was no association between symptom burden and confidence in the cardiologist.

Patients’ views on the level of AI involvement (Q4-Q7)

About a third (38.1%) of respondents would be happy (Q4 = 4 or 5) for their doctor to use AI in deciding treatment, while 30.9% would be unhappy. The remaining 30.9% were neutral (Q4 = 3). Unhappiness significantly increased to 61.8% if AI creates the treatment plan without doctor input (Q5) (p<0.001), and 20% were neutral. If AI adjusted their treatment plan from home and they saw a cardiologist less frequently, 60% percent would be unhappy (Q6 = 1 or 2) and 18.2% neutral. This is also a significant increase in unhappy patients compared to Q4 (p<0.001). If AI is better at improving their HF than doctors, 49% said they would still be unhappy for AI to create their treatment plan, while 32% said they would be happy (Q7).

Opinions in Q4 to Q7 showed no significant association with age, sex, education, previous experience with AI, symptom burden (Q1) or satisfaction with current follow-up (Q2). Patients who have a smartphone had 2.6-fold higher odds of reporting greater acceptance of AI making home treatment adjustments compared with non-owners (OR=2.63, 95% CI 1.19-5.77, p=0.017); however, subgroup sizes were imbalanced (78 users vs. 32 non-users). As such, the effect estimate should be interpreted cautiously.

Overall trust in AI versus cardiologist (Q8-Q10)

Most patients feel more confident if their diagnosis (80.9%) or treatment plan (84.6%) was made by a cardiologist compared to AI, with 15.5% and 12.7% feeling neutral respectively. Furthermore, if a cardiologist and AI came up with different treatment plans, 97.3% would trust the cardiologist over AI. These opinions have no significant association with age, sex, education level, experience with AI, smartphone ownership, how troublesome the HF (Q1) is and satisfaction with current follow-up (Q2).

## Discussion

In this study of patients with HF, satisfaction with current follow-up and confidence in their cardiologist’s treatment plan were generally high, with over 90% of respondents expressing positive views. Satisfaction with follow-up showed a graded association with symptom burden. Compared with patients whose HF symptoms were “not troublesome at all”, the odds of reporting greater satisfaction were substantially lower among those with very troublesome symptoms (Q1 = 1: OR = 24.9, 95% CI 1.38-449.8, p = .029), troublesome symptoms (Q1 = 2: OR = 11.2, 95% CI 1.27-98.9, p = .030), and slightly troublesome symptoms (Q1 = 4: OR = 17.3, 95% CI 1.60-188.9, p = .019). However, wide confidence intervals indicate limited precision. In contrast, confidence in the cardiologist’s treatment plan was not associated with symptom burden or demographic variables, suggesting that trust in the treating physician is relatively stable and less influenced by current health status. As a cross-sectional observational study, the relationships reported between symptom burden, satisfaction, and trust should be interpreted as associations, rather than causal effects. Prior work in chronic disease populations has similarly shown that while symptom burden shapes quality-of-life and satisfaction ratings, interpersonal trust in clinicians is largely independent of disease severity [27]. Trust in physicians relative to AI appears very strong: if cardiologists and AI were to come up with different treatment plans, 97.3% would trust the cardiologist over AI (Q10).

When exploring attitudes toward AI in clinical decision-making, we found mixed opinions. Over a third (38.1%) of participants were comfortable with their doctor using AI to guide treatment (Q4), but support declined substantially to 18.2% of patients when AI was described as acting without physician input (Q5) (p<0.001) or remotely adjusting treatment plans with fewer in-person visits (21.8%) (p<0.001). Even when AI was framed as outperforming doctors in improving outcomes (Q7), nearly half of the respondents remained unhappy with its use (49.1%), but 10.1% were neutral.

This pattern contrasts with findings from a systematic review by Young et al., which concluded that most patients and members of the public were supportive of AI, particularly in contexts such as symptom assessment and remote monitoring of chronic conditions [10]. Our more reserved results may reflect the unique context of HF care, which often involves an older patient population, higher levels of comorbidity, and a need for close, ongoing relationships with clinicians [23,28]. These factors may heighten the importance of human oversight and help explain the preference for clinician involvement observed in our sample. Similarly, respondents to the survey by Witkowski et al. were more comfortable with the use of AI when it is not associated with doctor-patient relationships, and the ‘human touch’ was an important factor [29]. This may explain why support for AI decreased when fewer in-person visits were suggested. Our findings also contrast with those of Jutzi et al. [30], who reported greater acceptance of AI when its accuracy exceeded that of physicians, and Zhang et al., who concluded that performance expectancy, the perceived benefit of a technology, is a major determinant of intention to use health technologies [31]. Importantly, Jutzi et al. focused on diagnostic accuracy, while Zhang et al. examined diabetes management apps in populations already familiar with such tools. In contrast, our questionnaire asked about treatment plan and our sample of HF patients had limited prior exposure to AI or digital health apps, which may explain their more cautious stance. We were unable to account for several factors that may influence both technology acceptance and satisfaction with care, including socioeconomic status, health literacy, internet quality, and access to digital devices beyond smartphones. These unmeasured variables may have confounded some associations and should be examined in future, larger studies.

A notable finding in our study is that smartphone ownership appears to be associated with greater willingness to accept remote AI adjustments with less in-person appointments (OR=2.63, 95% CI 1.19-5.77, p=0.017). This suggests that digital familiarity may reduce apprehension towards AI-enabled care. One plausible mechanism is that routine use of digital devices (such as smartphones) builds technology self-efficacy - the belief that one can successfully use digital tools - and digital health literacy [32]. In UTAUT framework, this feeds into effort expectancy and performance expectancy: patients who feel confident with technology perceive AI tools as easier to use and more likely to help, which in turn increases acceptance and intention to use [33]. Bol et al. concluded that digital health literacy should be considered a prerequisite of mobile health app use in general [34]. Digital self-efficacy and literacy reduce technophobia and internet anxiety, further lowering psychological barriers to engaging with AI-enabled care [34,35].

Interestingly, in our cohort, self-reported prior experience with AI did not predict attitudes to AI-enabled HF care, whereas smartphone ownership did. This may reflect the difference between general AI exposure (for example, hearing about AI in the media or using consumer AI tools) and practical digital capability in managing health. Prior AI exposure in non-clinical domains may do little to improve digital health literacy or confidence in using health apps, whereas regular smartphone use directly supports skills relevant to telemonitoring and app-based HF management. Studies of digital mental health and mobile health apps similarly suggest that trust, perceived usefulness and health-specific digital literacy are stronger drivers of adoption than generic familiarity with AI or technology [32]. This may explain why smartphone ownership, but not prior AI exposure, emerged as the more informative marker of readiness for AI-enabled HF follow-up in our sample. Similar associations have been reported by Leigh et al. and Bertolazzi et al.; although Kauttonen et al. found an inverted U-shaped relationship between AI knowledge and attitudes [36-38]. These findings suggest that successful implementation of AI in HF care may depend not only on technical accuracy, but also on supporting digital confidence and providing clear communication about clinical oversight. AI-driven technologies for patients with HF, such as HearO and Linea, offer compelling opportunities for timely intervention and remote care monitoring. HearO, for example, uses a smartphone app to analyse patients’ voice recordings and leverages algorithms to detect signs of fluid accumulation or decompensation in outpatient HF patients [8,39]. Linea integrates AI-enabled remote biometric and symptoms tracking, medical records and SMS-based support to enable early detection and intervention to reduce hospitalisations [9]. However, both solutions depend on smartphone ownership, digital literacy, reliable connectivity and regular engagement: requirements that risk excluding patients who are older, less educated, socioeconomically disadvantaged or live in areas with poor digital infrastructure. Unless implementation strategies actively address these access and literacy gaps, such technologies may inadvertently widen existing health-inequities in HF care. Policies and deployment frameworks should therefore prioritise inclusive access (e.g. provision of devices, digital-literacy support, multilingual interfaces) and align AI roll-out with clinician-led, patient-facing care teams to ensure vulnerable subgroups are not left behind.

No significant associations were found between age, sex or education level and patients’ satisfaction with current service, physician trust or AI attitudes in our cohort. There are mixed conclusions regarding how and whether these factors influence attitudes towards AI. Fritsch et al. reported that older patients, women and those with lower education were more cautious of AI, although their cohort had a higher proportion of participants who had read or heard about AI before [13]. Esmaeilzadeh reported that men, higher education and familiarity with AI were positive indicators for intention to use AI healthcare tools, although age had no association [14].

Overall, our results underscore the complexity of trust and adoption in medical AI. They highlight that while patients recognise potential benefits of AI, trust in clinicians remains paramount, particularly in conditions like HF, where treatment decisions are complex and deeply personalised. These findings align with prior research showing that interpersonal trust in healthcare is rooted in perceived empathy, accountability, and professional experience, qualities not easily attributed to AI [12,40].

A key strength of this study is its focus on patients with HFrEF, a group not previously studied with respect to attitudes toward AI, despite being a population where AI-enabled tools are increasingly being developed. Differences in what the existing literature reports as factors which influence AI attitudes highlight the need for developers to ensure suitable research is done on their target groups’ unique views on AI.

Our sample size was small and drawn from a single centre, which limits generalisability. The older age distribution and relatively low prior exposure to digital health tools may have biased responses toward caution regarding AI. Attitudes were measured through hypothetical scenarios rather than real-world interactions with AI, and responses may therefore differ from those observed in practice. Future research should investigate how HF patients engage with AI-enabled tools in real-world clinical settings. Larger, multicentre studies could clarify whether attitudes observed here are consistent across different populations and healthcare systems. This study relied on a questionnaire comprising self-reported perceptions. Question wording, scenario framing, and social desirability bias may all have influenced responses. Exploring how trust in AI may change with repeated exposure, transparent communication of its role, and clear evidence of clinical benefit will also be essential for guiding responsible implementation.

## Conclusions

Patients with HFrEF in this study expressed high satisfaction with their current follow-up and strong trust in their cardiologists, with confidence in physicians largely independent of symptom burden. While a third were comfortable with AI supporting treatment decisions, support declined sharply when AI was described as acting autonomously or reducing in-person clinician contact, indicating that trust in human oversight remains central to patients’ sense of safety. A distinctive contribution of this study is the demonstration that attitudes toward AI were shaped not by age, sex, or prior experience with AI, but by digital familiarity. In particular, greater acceptance among smartphone users suggests a role for digital self-efficacy rather than generic AI exposure in determining readiness for AI-enabled care. These findings highlight that AI solutions in HF management are most likely to be acceptable when designed to augment rather than replace clinicians, when supported by digital-literacy pathways, and when deployed in ways that do not disadvantage patients with limited access to digital technology. By identifying patient-centred determinants of AI acceptance within a HFrEF population, this study provides groundwork for equitable and clinically integrated implementation strategies and offers directions for future validation in larger and more diverse HF cohorts.

## Appendices

Literature review for designing the questionnaire

1.     Research identified through PubMed search. Keywords: “patient” AND (“perspective” or “attitude” or “perception”) AND (“artificial intelligence” or “AI”). Filters: years 2020-2024. (n=2826)

2.     Abstracts screened (n=86).

3.     Records reviewed in full (n=61).

Participant information sheet

Dear Patient

We are inviting you to participate in a survey. We are inviting you because you have a condition called heart failure. The survey will ask you what you think about heart failure and artificial intelligence (AI).

Artificial intelligence (AI) means “smart” computers or machines that are trained to think like humans can.

We want to know what you think about this because AI may have a role in diagnosing and treating heart failure in the future.

We are asking a lot of people with heart failure if they wish to take part in this survey.

The answers you give on the survey is anonymous. This means we won’t know it is you who gave these answers.

If you don’t want to complete the survey, you don’t have to and it will not affect any future treatment/care you may receive.

There is no benefit or harm that will come to you from completing the survey.

The results will be stored securely on the Trust hospital systems and be shared with the Cardiologist Team and the anonymous data may be used for a paper to be published in a medical journal.

If you have questions about the survey, please ask.

Questionnaire

Age:

Sex:

Education

___ Primary/secondary

___ Diploma

___ Bachelor’s degree

___ Post-graduate degree

Do you have a smartphone?

___ Yes

___ No

Experience of AI

___ Work or study in field related to AI

___ Understand how AI functions

___ Have researched the term AI

___ Have heard of the term AI

___ Do not know AI

Q1: How troublesome is your Heart Failure?

___ 1 - Not troublesome at all

___ 2 

___ 3

___ 4

___ 5 - Very troublesome

Q2: How satisfied are you with the follow-up you are getting for your heart failure?

___ 1 - Not satisfied at all

___ 2 

___ 3

___ 4

___ 5 - Very satisfied

Q3: How confident do you feel about the specialist doctor’s (cardiologist) treatment plan for your heart failure?

___ 1 - Not confident at all

___ 2 

___ 3

___ 4

___ 5 - Very confident

Q4: How would you feel if your doctor uses artificial intelligence to decide what treatment you should get for your heart failure?

___ 1 - Not happy at all

___ 2 

___ 3

___ 4

___ 5 - Very happy

Q5: How would you feel if artificial intelligence creates your heart failure treatment plan without any doctor being involved in the decision?

___ 1 - Not happy at all

___ 2 

___ 3

___ 4

___ 5 - Very happy

Q6: How would you feel if artificial intelligence decide and adjust your treatment plan from home and you see a cardiologist in person less frequently

___ 1 - Not happy at all

___ 2 

___ 3

___ 4

___ 5 - Very happy

Q7: How would you feel if artificial intelligence creates your treatment plan if artificial intelligence is better at improving your heart failure than a doctor

___ 1 - Not happy at all

___ 2 

___ 3

___ 4

___ 5 - Very happy

Q8: Do you agree or disagree: I feel more confident in my diagnosis when it is made by a cardiologist compared to artificial intelligence

___ 1 - Strongly disagree

___ 2 

___ 3

___ 4

___ 5 - Strongly agree

Q9: Do you agree or disagree: I feel more confident in my treatment plan when it is made by a cardiologist compared to artificial intelligence

___ 1 - Strongly disagree

___ 2 

___ 3

___ 4

___ 5 - Strongly agree

Q10: If a cardiologist and artificial intelligence programme come up with different treatment plans for your heart failure, whose plan would you trust more?

___Cardiologist

___ Artificial intelligence

Credits: Sirindhra Suepiantham, Ravish Katira

Frequency tables

**Table 2:** Frequency tables.

Q1		
Likert	Frequency	Percent
1		
2		
3		
4		
5		

Q2		
Likert	Frequency	Percent
1	2	2
2	0	0
3	5	5
4	14	13
5	89	81

Q3		
Likert	Frequency	Percent
1	1	1
2	0	0
3	5	5
4	26	24
5	78	71

Q4		
Likert	Frequency	Percent
1	22	20
2	12	11
3	34	31
4	16	15
5	26	24

Q5		
Likert	Frequency	Percent
1	54	50
2	14	13
3	22	20
4	10	9
5	10	9

Q6		
Likert	Frequency	Percent
1	46	42
2	20	18
3	20	18
4	9	8
5	15	14

Q7		
Likert	Frequency	Percent
1	34	31
2	20	18
3	21	19
4	19	17
5	16	15

Q8		
Likert	Frequency	Percent
1	4	4
2	0	0
3	17	16
4	18	16
5	71	65

Q9		
Likert	Frequency	Percent
1	1	1
2	2	2
3	14	13
4	21	19
5	72	67

Q10		
	Frequency	Percent
Cardiologist	107	97
AI	3	3
